# Assessment of Anxiety among People with Various Physical Activity Levels during Lockdown in Karachi, Pakistan

**DOI:** 10.1055/s-0043-1770934

**Published:** 2023-07-12

**Authors:** Noor ul Ain, Muriam Arif, Mariam Sultan Dar, Umm e Habiba, Mahjabeen Shah, Uzma Naseeb

**Affiliations:** 1 Jinnah Sindh Medical University, Karachi, Pakistan; 2 Department of Biochemistry, Jinnah Sindh Medical University, Karachi, Pakistan

**Keywords:** physical activity, anxiety, COVID-19, psychological well-being, mental health

## Abstract

**Background** The purpose of this study is to make it easier to counsel the general public on how to promote their psychological health and better lifestyle by boosting their physical activity in the event of more devastating future waves of coronavirus disease 2019. Coronavirus disease is a viral infection first reported in Wuhan, China, which led to a worldwide pandemic and lockdown. In addition to physical health, the uncertainty of situation and fear of infection have further affected the mental health of the individuals. Lockdown has also halted the physical activity of people further adding into the stress and anxiety.

**Methods** A sample size of 376 was required, but a convenient sampling technique collected 400 responses due to overwhelming Participation. A well-structured questionnaire was used for data collection, which contained sections such as a demographics section. We used the International Physical Activity Questionnaire scale to measure various physical activity levels and the generalized anxiety disorder scale to measure anxiety levels, because of their considerate validity and feasibility. Later both of our variables were compared to find out the correlation of anxiety levels with different physical activity levels.

**Results** Mean Anxiety and its prevalence rates in the sample were 7.32 ± 5.767 and 33%. On performing Pearson correlation, increasing age was found to be a risk factor for Anxiety. One-way analysis of variance showed a significant difference between Physical Activity and Anxiety. In the post-hoc Tukey test, a considerable difference in anxiety level was found between people with no physical activity and vigorous physical activity.

**Conclusion** During the lockdown period, the mean anxiety was found to be 7.0 ± 5.56 with prevalence rate of 33% among the people with no, low, moderate or vigorous physically active levels was found to be associated with better perceived mental health status which suggests the need to promote physically active style to cope with anxiety and awareness sessions to be conducted especially among those who could not maintain a physically active lifestyle. Also, among the general population e-Health programs could provide an appropriate way to promote a physically active lifestyle.

## Introduction

The coronavirus infection is a viral infection that was reported in Wuhan City, Hubei Province, China, in late December 2019. 1 Coronavirus disease 2019 (COVID-19) can cause a variety of symptoms. Symptoms might range from mild-to-severe acute respiratory distress syndrome with failure of many organs. 2 Because this virus is human-to-human transmissible, 1 it has spread rapidly outside of China. In March 2020, the World Health Organization designated it a significant global outbreak. 3 The death toll continues to rise, forcing many countries to impose social distancing and lockdown to contain the virus (severe acute respiratory syndrome coronavirus 2). 4 China became the first to set the Wuhan lockdown on the 25th day after the initial infection, and by the end of January, 16 more cities, followed by the rest of the world, culminating in what is now known to be the world's most extensive quarantine. 3 5 In Pakistan, the first city infected with the virus was Karachi, from where the first confirmed incidence occurred on February 26, 2020, and on March 23, 2020, Pakistan implemented a lockdown to control the disease's spread. 6

Many studies indicate that aside from the medical dangers, the pandemic 7 and the efforts attempted to limit the spread, 8 whether or not it is successful in containing the outbreak, have wreaked havoc on people's mental health and social lives. 9 10 Anxiety, depression, and stress are some of the psychological reactions 11 . One study of 253 individuals from one of the COVID-19 pandemic's hardest-hit regions showed that 1 month following the epidemic, China had a 7% rate of psychological stress. 12 A study in Turkey showed that the average subjects' anxiety and depression ratings were 6.8 and 6.7 with a standard deviation of 4.2 for both anxiety and depression, respectively. 10 The overall degree of anxiousness was 8.61 with a standard deviation of 6.95, and the degree of anxiety symptoms was average in 49.1% cases, severe in 9.3%, and very severe in 9.8% of cases, according to the research conducted in Iran. 13 Since the pandemic in Hong Kong, 25.4% of people have claimed that their mental health has deteriorated. 14 According to a study conducted in Ireland, generalized anxiety disorder (GAD) was detected in 20.0% (95% confidence interval = 17.55, 22.41) of the participants, while 22.8% of the participants were negative. 15 The study in India found that the individuals had severe anxiety. More than 80% were preoccupied with COVID-19-related thinking. More than 80% of those respondents said that they needed mental health care. 16 Anxiety and depression were found to be prevalent in 43% of Pakistani frontline physicians. 17 A study in Pakistan showed that the students had an average (43%), mild (20%), moderate (13%), and severe (22%) degrees of anxiety, according to the findings. 18 Another study in Pakistan found 92.3% of nurses suffered from light to extreme anxiety, with females experiencing significantly greater anxiety levels. 19

Previous research suggests that regular physical activity (PA) has a beneficial effect on anxiety's pathophysiological processes. 20 21 Consequently, the lockdown is a crucial element in anxiety development because, for two reasons, it is likely to have affected people's typical levels of PA. First, the lockdown halted all nonessential mobility, with gym closures, public movement restrictions, and local transportation among them, all of which have stirred the everyday routine into turmoil. Second, the fear of becoming infected with the virus may make the public less reluctant to depart from their homes and engage in routine activities. 22 So, people who cannot adapt to a new lifestyle become increasingly burdened, resulting in anxiety. 21

In the world of modern medicine, the advantages of daily PA on anxiety are inevitable. 20 PA has an antianxiety impact that is independent of age and geographical region. 23 According to another research published recently, PA's light, moderate, and vigorous intensities are related to well-being and a reduction in the negative psychosocial impact of confinement when in lockdown due to COVID-19. 24 However, evidence for which level is most effective in reducing anxiety is limited, and related theoretical research lags practice. This research aims to understand the association between different PA levels and the preponderance of anxiety during this pandemic lockdown in Karachi.

The purpose of this study is to make it easier to counsel the general public on how to promote their psychological health and better lifestyle by boosting their PA in the event of more devastating future waves of COVID-19. We hypothesized that people with decreased PA levels are more prone to develop anxiety than those who engage in vigorous PA.

## Methodology

### Study Design

We carried out cross-sectional research with a convenient sample technique. The targeted population included people of different genders, diverse occupational backgrounds (students, teachers, office employees, and business owners), and other educational levels (graduate and undergraduate). And the association between anxiety level and PA level was measured amid the COVID-19 pandemic.

### Data Collection and Sample

The sample size was 376, which was calculated through openEpi version 3, having a population of 20,000,000. And we kept the confidence level at 95% to keep the study reliable, with error limited to 5%, and the anticipated frequency was kept as 57.7%, according to a study conducted in Karachi. 25 Data was obtained throughout the lockdown time from June 8, 2021 to July 3, 2021, through a well-structured online google e-form using a convenient sample technique. E-form was disseminated through email, WhatsApp, and various social media platforms. And consent was also taken through the same e-form.

### Rating Instruments

A well-structured questionnaire was our rating instrument, which was divided into sections for study variables. It started with demographics in which we inquired age, gender, occupation, height, and weight to correlate with our study's dependent variables. It also contained a social awareness segment having multiple choice questions regarding COVID-19 and anxiety prevalent by the ongoing pandemic. We used the International Physical Activity Questionnaire (IPAQ) scale for measuring PA levels 26 and GAD7 27 28 for anxiety levels among our targeted population amid COVID-19 pandemic. Then we compared anxiety levels with PA to find a correlation between PA and anxiety levels.

### IPAQ Scale

The international PA short form is robust and effectual for evaluating PA levels in people aged 18 to 60. 29 30

We asked about four types of PAs using IPAQ: vigorous PAs (carrying heavyweight, etc.), moderate PAs (lifting, lightweight, etc.), mild PAs (walking), and no PAs (sitting). We then graded them in four categories: no, low, moderate, and vigorous PAs.

### GAD-7

GAD-7 is a seven-item measure that asks seven questions about a primary symptom of a GAD. 31 Additionally, it enables you to assess different levels of anxiety in your target group. It has a 0.83 Cronbach's alpha value. Hence, it is a reliable and credible scale. 32 GAD-7 was used to evaluate different anxiety levels in our sample. A total score of 10 showed that there was a possibility of anxiousness. 14

### Data Analysis

SPSS software version 20.0 was used for data analysis with a 95% confidence interval and a statistical significance level of 0.05 as the statistical analysis threshold. A significant difference between anxiety level and PA was measured using one-way analysis of variance (ANOVA). Once this was determined, the post-hoc Tukey test was used to determine where the actual difference lies among different categories of PAs.

### Ethical Consideration

Before conducting the research, approval was received from the Institutional Review Board of the Jinnah Sindh Medical University, Karachi. Subjects gave consent after being informed about the study's purpose; no parental or guardian consent was required because the participants were over the age of 18. Participants actively engaged, understanding that their supplied data would be used wisely and that any personal information they shared would be kept private.

## Results

During the Lockdown in Karachi, a study was undertaken on assessing anxiety among people with varying levels of PA. The study was conducted online, and 800 people were contacted via WhatsApp and Facebook. Although our sample size was 376, 400 people out of 800 replied and finished the online questionnaire, resulting in a 50% response rate. The study only included persons who were between the ages of 18 and 60. The average age of the participants was 24.66 ± 7.29 years. Females ( *n* = 284, 79%), undergraduates ( *n* = 278, 69.5%), and students ( *n* = 310, 77.5%) made up the majority of the participants. Anxiety was determined to be 7.32 ± 5.767 on average in the sample. During the lockdown period, the prevalence of anxiety (GAD-7 score 10 or greater) was determined to be 33%. There were 86 (21.5%), 175 (43.75%), 45 (11.25%), and 94 (23.5%) participants with no, low, moderate, and vigorous PA levels, respectively ( Table 1 ).

**Table 1:** Demographic characteristics of respondents

Sociodemographics	*n* , %
** Age groups (years), M ± SD** 18–26 27–36 37–46 47–60	24.66 ± 7.29 342 (85.5%) 25 (6.3%) 17 (4.2%) 16 (4%)
** Gender** Male Female	116 (21%) 284 (79%)
** Education level** Not formally educated Primary Matriculation Intermediate Undergraduate Graduate Postgraduate	4 (1%) 2 (0.5%) 5 (1.3%) 32 (8%) 278 (69.5%) 55 (13.7%) 24 (6%)
** Occupational level** Business owner Government sector employee Homemaker Private sector employee Retired Self-employed Student Unemployed	7 (1.7%) 10 (2.5%) 7 (1.7%) 36 (9%) 1 (0.2%) 16 (4%) 310 (77.5%) 13 (3.2%)
** GAD score** No anxiety Mild anxiety Moderate anxiety Severe anxiety	38.50% 28.50% 19.50% 13.50%
** Physical activity levels** No physical activity Low physical activity Moderate physical activity Vigorous physical activity	21.50% 43.75% 11.25% 23.50%

Abbreviations: GAD, generalized anxiety disorder; M, mean; SD, standard deviation.

*n* = 400.

We used Pearson correlation to look at the relationship between age and anxiety, and it was discovered that a weak positive correlation exists between age and anxiety ( *p* = 0.095; ( Table 2 ). This shows that as one variable increases, the other increases as well although the relationship is not too strong. So, as people get older, their anxiety levels also tend to rise.

**Table 2:** Pearson correlation for anxiety and age

		Mean age	GAD-total
Mean age	Pearson correlation	1	0.095
	Sig. (2- tailed)		0.058
	*n*	400	400
G	Pearson correlation	0.095	1
	Sig. (2- tailed)	0.058	
	*n*	400	400

Abbreviation: GAD, generalized anxiety disorder.

There was a nonsignificant difference in anxiety level with the progressive increase in education level ( *p* = 0.633) and a nonsignificant difference in anxiety with occupation level ( *p* = 0.724), which was calculated using the one-way ANOVA test. There was also a statistically significant difference in anxiety levels between no, low, moderate, and vigorous PA, with *p* = 0.027 determined using the one-way ANOVA test ( Table 3 ). To see where the difference was, we used a post-hoc Tukey test. A significant difference of *p* = 0.02 and a mean difference of M = − 2.436 was found between persons who did not participate in any physical exercise and people who engaged in vigorous PA, indicating that anxiety levels decreased with vigorous PA ( Table 4 ).

**Table 3:** One-way ANOVA of education, occupation, physical activity levels with anxiety level

		Sum of squares	df	Mean square	F	p-Value
Education level	Between groups	144.436	7	24.073	0.721	0.633
	Within groups	13,126.961	393	33.402		
	Total	13,271.398	400			
Occupation level	Between groups	162.621	7	27.104	0.607	0.724
	Within groups	13,126.961	393	44.628		
	Total	7,615.477	400			
Physical levels anxiety	Between groups	302.932	3	100.977	3.089	a 0.027
	Within groups	12,975.617	397	32.684		
	Total	13,278.549	400			

Abbreviation: ANOVA, analysis of variance.

*n* = 400.

a *p* -Value ≤ 0.05.

**Table 4:** Multiple comparisons of physical activity levels for generalized anxiety disorder scale Tukey HSD

(I)IPAQ-group	(J)IPAQ-group	Mean difference(I-J)	Standard error	*p* -Value
No physical activity	Low physical activity	1.447	0.761	0.229
	Moderate physical activity	0.729	1.059	0.902
	Vigorous physical activity	2.436	0.861	a 0.025
Low physical activity	No physical activity	–1.447	0.761	0.229
	Moderate physical activity	–0.718	0.958	0.877
	Vigorous physical activity	0.989	0.733	0.532
Moderate physical activity	No physical activity	–0.729	1.059	0.902
	Low physical activity	0.718	0.958	0.877
	Vigorous physical activity	0.989	1.039	0.356

Abbreviations: HSD, honest significant difference; IPAQ, International Physical Activity Questionnaire.

Dependent variable = Generalized anxiety disorder scale.

Post-Hoc Tukey test.

a *p* -Value ≤ 0.05.

## Discussion

Globally, the COVID-19 epidemic has caused a significant hazard to healthcare institutions, psychological well-being, and the financial sector. COVID-19 has resulted in a substantial increase in mortality and frequent quarantines and lockdown measures, all of which have had an adverse impact on people's mental health, frequently neglected, and underemphasized. 33 This study examines anxiety in people who engage in various PAs. In this research, we discovered the average level of anxiety during COVID-19 lockdown that is 7.0 ± 5.56. This finding is in contrast with the study conducted before the COVID-19 pandemic at Agha Khan Hospital in Karachi, Pakistan, in which mean anxiety level was found to be 5.7 ± 3.86. 34

Furthermore, our findings show that as people's age increased, so did their anxiety levels. This could be attributed to a gradual decline in PA as people get older, which leads to an increase in worry. This finding is based on statistical analysis of cross-sectional research data that showed a weak positive correlation between age and anxiety. Further research and different study designs are needed to establish the credibility of the result. Other researchers have found that younger people are the most exposed to the psychological consequences of the epidemic due to increased social media exposure and uncertainty about the current situation. 35 36 Furthermore, we discovered that persons who engage in moderate or strenuous PA have lower anxiety levels than those who do not participate in PA.

This result is similar to a study by Pieh et al, which discovered that increasing the duration and extremity of PA reduced depression and anxiety in males, adults, and children. 37 Another study by Anyan et al evaluated anxiety levels in different subgroups of PA and found that the subgroup with higher PA had the lowest anxiety symptoms amidst the pandemic. 11 COVID-19-mediated lockdown is linked to negative emotions such as despair, rage, frustration, and a significant rise in anxiety. 38 According to studies, being less physically active is related to having more negative feelings. As a result, exercising or engaging in any form of mild-to-moderate activity during this pandemic can help to alleviate the psychological effects. 39 According to research, moderate to intense activity can increase blood flow to a part of the brain involved in stress response management. 40 Previous research has suggested that the synthesis of certain neurotransmitters, 41 neurotropic factors, activation of anti-inflammatory processes, and enhanced organ system functioning may play a role in this protective impact against anxiety. 42 We found no difference in levels of anxiety between males and females in our research. As opposed to this finding, analysis from Karachi found that females have the highest levels of anxiousness. 43

This study is likely the first to look at anxiety with varying amounts of PA in Karachi, Pakistan. Because the data for this study was acquired during a complete lockdown in Karachi, the results' accuracy can only be guessed. This study emphasizes the significance of PA in sustaining psychological well-being during the epidemic.

There are several limitations of this study as well. Firstly, convenience sampling was used, and participants were contacted via social media, resulting in selection bias. Second, because of the complete lockdown, we could not personally assess PA. Thus, we relied on the IPAQ questionnaire to determine PA levels. As a result, more study is urged to improve accuracy. Third, because this cohort was relatively reachable, the study was skewed toward the 18 to 26 age group and females.

## Conclusion

During the lockdown, the mean anxiety level was determined to be 7.0 ± 5.56, with a prevalence rate of 33%. High PA levels were linked to a better perception of psychological health, implying the need to hype a physically active anxiety-relieving lifestyle during the pandemic lockdown. Increasing anxiousness was also associated with advancing age, suggesting the need for awareness sessions, particularly among those unable to maintain a physically active lifestyle as their age progresses. Community-based eHealth programs could also be an effective strategy to encourage a physically active lifestyle among the general public.
